# Diagnostic Yield of Extended Cardiac Patch Monitoring in Patients with Stroke or TIA

**DOI:** 10.3389/fneur.2014.00266

**Published:** 2015-01-12

**Authors:** Christie E. Tung, Derek Su, Mintu P. Turakhia, Maarten G. Lansberg

**Affiliations:** ^1^Department of Neurology, Stanford University, Palo Alto, CA, USA; ^2^Division of Cardiovascular Medicine, Department of Medicine, Stanford University, Palo Alto, CA, USA; ^3^Veterans Affairs Palo Alto Health Care System, Palo Alto, CA, USA

**Keywords:** stroke, telemetry, atrial fibrillation, cardiac monitoring, arrhythmias, cardiac, TIA, SVT

## Abstract

**Background:** It is important to evaluate patients with transient ischemic attack (TIA) or stroke for atrial fibrillation (AF) because the detection of AF changes the recommended anti-thrombotic regimen from treatment with an antiplatelet agent to oral anticoagulation. This study describes the diagnostic yield of a patch-based, single-use, and water-resistant 14-day continuous cardiac rhythm monitor (ZIO Patch) in patients with stroke or TIA.

**Methods:** We obtained data from the manufacturer and servicer of the ZIO Patch (iRhythm Technologies). Patients who were monitored between January 2012 and June 2013 and whose indication for monitoring was TIA or stroke were included. The duration of monitoring, the number and type of arrhythmias, and the time to first arrhythmia were documented.

**Results:** One thousand one hundred seventy-one monitoring reports were analyzed. The mean monitor wear time was 10.9 days and the median wear time was 13.0 days (interquartile range 7.2–14.0). The median analyzable time relative to the total wear time was 98.7% (IQR 96.0–99.5%). AF was present in 5.0% of all reports. The mean duration before the first episode of paroxysmal AF (PAF) was 1.5 days and the median duration was 0.4 days. 14.3% of first PAF episodes occurred after 48 h. The mean PAF burden was 12.7% of the total monitoring duration.

**Conclusion:** Excellent quality of the recordings and very good patient compliance coupled with a substantial proportion of AF detection beyond the first 48 h of monitoring suggest that the cardiac patch is superior to conventional 48-h Holter monitors for AF detection in patients with stroke or TIA.

## Introduction

It is important to evaluate patients with stroke or transient ischemic attack (TIA) for atrial fibrillation (AF), because the detection of AF changes the recommended anti-thrombotic regimen from treatment with an antiplatelet agent to treatment with oral anticoagulation (1).

Atrial fibrillation is a well-recognized cause of stroke and TIA and recent studies of extended cardiac rhythm monitoring demonstrate that AF may be responsible for a greater proportion of unexplained strokes than previously realized. In a systematic review and meta-analysis of 32 studies of patients with stroke or TIA, the overall detection rate of AF was 11.5% (2). In the recent CRYSTAL-AF study, AF was detected in 29% of cryptogenic stroke patients who underwent 1 year of cardiac monitoring with an implantable cardiac loop recorder (3). The detection of supraventricular tachycardia (SVT) may be important as well as it is associated with the development of AF and an increased risk of stroke even in patients without AF (4–6). These results suggest an important role for extended cardiac monitoring in the evaluation of patients with stroke or TIA.

Because of memory and technical limitations, ambulatory cardiac monitoring has historically provided either short-term (up to 48 h) continuous monitoring (Holter) or longer term intermittent monitoring (event or loop recorders). Longer term continuous monitoring has been limited by patient compliance, the analyzable wear time, and electrode skin irritation (7). A novel, patch-based, water-resistant cardiac patch rhythm monitor (ZIO Patch) can provide continuous cardiac monitoring for up to 14 days. A recent study in patients with cardiac indications concluded that the patch monitor has high patient compliance, high analyzable time, and incremental diagnostic yield beyond 48 h for all arrhythmia types (8). We investigated the diagnostic yield of the cardiac patch monitor for long-term continuous cardiac monitoring in a large nationwide sample of stroke and TIA patients.

## Materials and Methods

**Figure 1:**
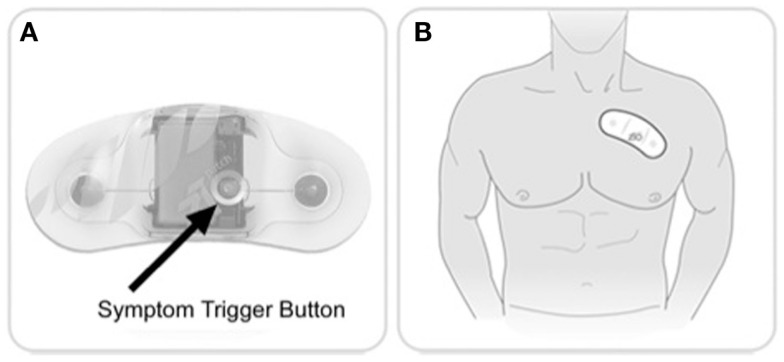
**Ziopatch cardiac monitor**. Zio Patch button and placement. **(A)** Patients can press a button on the Zio Patch to mark a symptomatic episode. **(B)** The device is placed over the patient’s left pectoral region. (Images courtesy of iRhythm Technologies, Inc., San Francisco, CA, USA.)

The self-adhesive cardiac patch was typically applied by trained technicians at the ordering clinic or hospital. A button could be pressed on the patch by the patient to annotate symptoms [Figure 1, Ref. (8)]. During the monitoring period the patient kept a diary of symptoms and annotations. After completion of the monitoring period, the patient mailed the device and diary to a central data processing center, where the data were analyzed by proprietary computer algorithms in conjunction with certified cardiac technicians and cardiologists to curate a clinical report.

All patients who underwent monitoring with the ZIO Service in the United States between January 2012 and June 2013 and whose indication for monitoring, entered as free text by the ordering provider, was stroke or TIA were included in this study.

We obtained the de-identified monitoring data from the data processing center (iRhythm Technologies Inc., San Francisco, CA, USA). This research was exempt from IRB review and approval as it did not meet criteria for human subjects research. All data were collected as part of routine clinical practice and the investigators could not ascertain the identity of the individual patients included in the study. The data were transferred after removal of all patient-, physician-, and site-level identifiers. The duration of monitoring, analyzable signal time, the number and type of arrhythmias, and the time to first arrhythmia were documented. The duration of monitoring was calculated as the total wear time, which was from the point of activation to the point of the last recorded analyzable signal. Analyzable signal time was defined as the proportion of the total wear time that the electrocardiographic (ECG) signal is interpretable (sufficiently free of noise). SVT was defined as four or more supraventricular ectopic beats and AF was defined as R–R irregularity for more than 30 s. AF was classified as either paroxysmal or chronic (100% of analyzable time). For patients with paroxysmal AF (PAF), the AF burden was calculated as the proportion of the analyzable signal time that consisted of AF. The cumulative diagnostic yield was calculated based on the method of Kaplan and Meier.

## Results

One thousand one hundred seventy-one monitoring reports were analyzed. The average patient age was 67.9 years and 55% of patients were men. The median wear time was 13.0 days [interquartile range (IQR) 7.2–14.0]. 97.1% of the patients wore the monitor for more than 48 h, 91.9% wore the monitor for more than 4 days, and 66.9% wore the monitor for more than 10 days. The mean analyzable time, expressed as a proportion of the total wear time, was 95.8%. The median analyzable time was 98.7% (IQR 96.0–99.5%).

**Figure 2:**
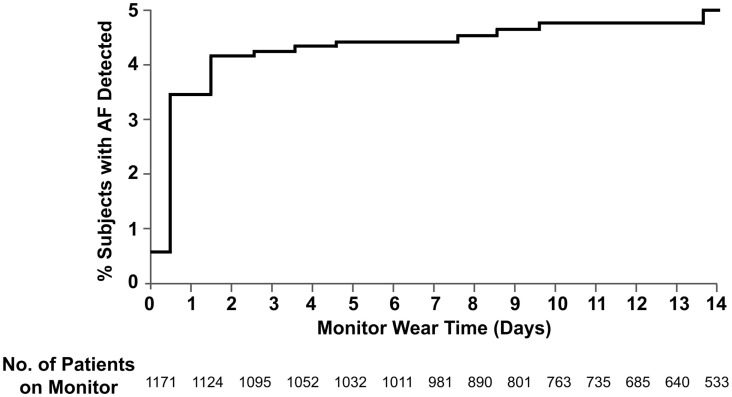
**Cumulative frequency of atrial fibrillation**. Shown is the probability of detection of atrial fibrillation in stroke and TIA patients with cardiac patch monitoring up to 14 days. The data are shown on an enlarged *y*-axis.

Using Kaplan–Meier statistics, the frequency of AF at 14 days was 5.0% (4.4% PAF and 0.6% chronic AF) (Figure 2). The mean duration before the first episode of PAF was 1.5 days and the median duration before the first episode was 0.4 days. 14.3% of first PAF episodes occurred after 48 h. The mean PAF burden was 12.7% of the total monitoring duration. The median PAF burden in hours was 0.33 h (IQR 0.11–1.33).

Supraventricular tachycardia of four beats or more was present in 70.2% of recordings. SVT of eight beats or more was present in 51% of recordings. ninety-nine percent of patients with SVT had no AF. The mean duration before the first SVT recording was 2.1 days. The median duration of the longest SVT recording was 5.9 s (IQR 3.3–10.6). The median number of beats in the longest SVT recording was 12 (IQR 7–22).

## Discussion

This is the first large-scale study of cardiac patch monitoring in patients with a history of stroke or TIA. It demonstrates that the cardiac patch, a novel device for detection of AF and other cardiac arrhythmias, is well tolerated and results in detection of AF in 5% of patients with stroke or TIA. A substantial proportion of patients with PAF had their first episode more than 48 h after the start of the monitoring period. These findings indicate that extended continuous monitoring with the cardiac patch monitor may lead to the identification of AF that would likely have been missed with traditional 48-h Holter monitoring.

The very high rate of SVT detection (70%) in this patient population with a history of stroke or TIA is noteworthy. However, the median duration of SVT was very short (6 s) and the significance of short episodes of SVT is unclear. A recent study of patients with pacemakers or implantable cardioverter-defibrillators showed that subclinical atrial tachyarrhythmia, a form of SVT, defined as an episode of rapid atrial rate (≥190 beats per minute) lasting more than 6 min, often preceded the development of clinical AF. It also showed a nearly 2.5-fold increased risk of stroke in patients with atrial tachyarrhythmias, regardless of the presence of AF (9). Further studies are needed to elucidate the association between short runs of SVT and stroke and to determine if anticoagulation is superior to antiplatelet therapy for secondary stroke prevention in certain patients with SVT (e.g., presence of long and/or frequent episodes). It should be stressed that detection of SVT is currently not an indication for oral anticoagulation in patients with a history of stroke or TIA.

Patch monitoring had very high patient compliance (median wear time 13.0 days) and analyzable time (98.7%). This might have resulted from the practical benefits of patch-based monitoring compared with traditional “Holter” monitors that have detachable leads, removable skin electrodes, and a separate recording unit. These benefits include its small size, its water resistance, and the absence of wired skin electrodes. Difficulty of use, skin infection or irritation, and disruption to the patient’s work, travel, and lifestyle have been reported as causes of reduced compliance in previous studies with lead-based extended monitors (7, 10). Wired monitors are also limited by motion artifact during exercise and other physical activity and must be removed before showering and contact with water.

There were several differences between our study and prior studies of cardiac monitoring. In contrast to prior post-stroke or TIA studies, which typically obtained detailed data on a relatively small number of patients, our study included a national sample of all stroke and TIA patients who underwent cardiac patch monitoring in the US during an 18-month time-period. Due to the retrospective design, however, we had limited clinical data on the subjects in the study. For example, we had no data on the presumed etiology of the strokes and TIAs.

Another limitation is confounding by indication. The clinical indication of stroke or TIA was based on free-text input from the ordering provider. It could be that clinicians preferentially ordered cardiac patch monitoring, as opposed to shorter term monitoring, when they suspected a low likelihood of arrhythmias. This could underestimate the true AF yield with cardiac patch monitoring in all-comers with stroke or TIA. Alternatively, if physicians preferentially ordered cardiac patch monitoring when they suspected a high likelihood of AF, the results could be an overestimation of the AF detection rate in all-comers.

The rate of AF detection in this study (5%) was lower than in most previous studies of cardiac monitoring in stroke and TIA patients. This may, in part, be explained by differences in the proportions of patients with cryptogenic strokes. The current study likely consisted of a more heterogeneous stroke and TIA population, including many patients with non-cryptogenic strokes, whereas most prior studies have preferentially enrolled patients with cryptogenic strokes. A meta-analysis of cardiac monitoring in patients with stroke or TIA has shown that the rate of AF detection is significantly higher in patients with cryptogenic strokes (15%) than in patients with non-cryptogenic strokes (11.5%) (2). In EMBRACE, a recent large multicenter randomized study of patients with cryptogenic stroke or TIA, AF was detected in 16% of patients after 30 days of cardiac monitoring (11).

Variation in monitoring duration may also have contributed to differences in the detection rate of AF. In our study, we found the highest rate of AF detection in the first days of monitoring and a marked decline in the yield of AF detection during the second week. These results suggest that a 2-week monitoring duration may be sufficiently long. However, the optimal duration of monitoring is not known and longer monitoring will likely increase the yield of AF detection. This is exemplified by the results of the CRYSTAL-AF study, which assessed the diagnostic yield of an implantable cardiac loop recorder during 3 years of monitoring. In this study, the yield of AF detection at 6, 12, and 36 months was 8.9, 12.4, and 30% (3). The increased yield of AF detection with multi-year cardiac monitoring should be weighed against the drawbacks associated with the use of an implantable cardiac loop recorder, including the need for an invasive procedure, cost, incompatibility with magnetic resonance imaging, and procedural risks such as infection.

Another limitation of our study is the lack of a direct comparison group. It is possible that a subset of the patients would have been diagnosed with AF even if they had no patch monitor, either during routine clinical follow-up or because they experienced symptoms from their PAF. A proposed study, comparing AF detection rates among stroke and TIA patients randomized to the cardiac patch monitor versus a traditional Holter monitor, will address this issue.

In sum, our data indicate that AF detection in stroke patients with a 14-day cardiac patch monitor is associated with excellent recording quality, high patient compliance, and a substantial proportion of AF detection that occurs beyond the first 48 h of recording. This suggests that cardiac patch monitoring will increase the detection of AF in stroke and TIA patients as compared to traditional 48-h Holter monitoring.

## Conflict of Interest Statement

Mintu P. Turakhia and Maarten G. Lansberg have received grant support from iRhythm, Inc. Mintu P. Turakhia is a consultant for Medtronic Inc., St Jude Medical Inc., and Daiichi-Sankyo. Christie E. Tung and Derek Su have no conflicts of interest to declare.
